# The in vitro efficacy of eye drops containing a bacteriophage solution specific for *Staphylococcus* spp. isolated from dogs with bacterial conjunctivitis

**DOI:** 10.1186/s13620-020-00175-x

**Published:** 2020-11-04

**Authors:** Renata Urban-Chmiel, Ireneusz Balicki, Katarzyna Świąder, Anna Nowaczek, Ewelina Pyzik, Dagmara Stępień-Pyśniak, Agnieszka Marek, Andrzej Puchalski, Andrzej Wernicki, Ewa Poleszak, Marta Dec

**Affiliations:** 1 SubDepartment of Veterinary Prevention and Avian Diseases, Institute of Biological Bases of Animal Diseases, Faculty of Veterinary Medicine, University of Life Sciences Lublin, Lublin, Poland; 2 Department and Clinic of Animal Surgery, Faculty of Veterinary Medicine, Lublin, Poland; 3 Department of Applied Pharmacy, Medical University of Lublin, Lublin, Poland

**Keywords:** Antibiotic resistance, Conjunctival diseases, Experimental medicine, Bacterial infections, Ophthalmology

## Abstract

**Background:**

The purpose of the study was to evaluate the in vitro antibacterial effect of experimental eye drops with bacteriophages in elimination of *Staphylococcus* spp*.* isolated from dogs with bacterial conjunctivitis.. The bacterial material was collected from dogs with independent clinical signs of bacterial conjunctivitis. *Staphylococcus* spp. were identified by phenotypic and genotypic methods (MALDI-TOF MS mass spectrometry). Antibiotic resistance was determined by the disc-diffusion method. Phage activity (Plaque forming units, PFU) was determined on double-layer agar plates. Phages with lytic titres > 10^8^ PFU were used to prepare eye drops. The stability of the antibacterial titre was evaluated for preparations stored in sealed bottles as well as after opening and reclosing.

**Results:**

The tests confirmed the occurrence of *Staphylococcus* spp. strains as etiological agents of bacterial conjunctivitis in dogs. A high percentage of strains were resistant to more than three antibiotics. The experimental phage eye drops used in the study exhibited 100% efficacy in vitro against the tested *Staphylococcus* isolates. Particularly noteworthy is the long duration of activity and constant antibacterial lytic titre of ≥10^8^ PFU/mL of two eye drop solutions, nos. 7 and 12, after the bottle had been opened (21 days) and after hermetically sealed packaging (28 days) at 4–8 °C.

**Conclusions:**

The results represent the first stage of research and require continuation in vivo. If positive effects are obtained in animals, the results can be used in applied research in humans and animals.

## Background

Bacterial conjunctivitis in dogs often caused by different Staphylococcus spp. strains is a frequently diagnosed health problem worldwide [1]. An important factor limiting the control and effective treatment of infections is the increasing multi-drug resistance of strains to the antibiotics used to treat them. Primary bacterial conjunctivitis is uncommon in dogs, rather it is often associated with other ophthalmic or systemic diseases [2]. Bacterial conjunctivitis should be diagnosed by comprehensive ophthalmic diagnostics, including the Schirmer test, Jones test, and corneal examination using a slit lamp [3, 4]. Diagnosis and monitoring may be facilitated by conjunctival cytology, which provides information regarding epithelial cell metaplasia, bacteria morphology, and staining characteristics – Gram-positive or negative [5–8]. In acute bacterial conjunctivitis, the dominant immune cells are neutrophils, with only a few mononuclear cells and degenerating epithelial cells. Bacterial cells also appear in the smear [3].

Symptoms of bacterial conjunctivitis include varying degrees of conjunctival redness, conjunctival oedema, and the presence of a purulent exudate of varying severity [5, 9–11]. In cases of bacterial conjunctivitis, a detailed ophthalmological examination is often followed by bacterial culture and susceptibility. The most common pathogens isolated from conjunctivitis in dogs are *Staphylococcus* spp., *Streptococcus* spp., *Bacillus* spp., *Pseudomonas* spp., *Corynebacterium* spp., and *Escherichia coli* [8, 9].

Therapy for bacterial conjunctivitis must be combined with treatment of the primary disease. In its acute form, it is limited to administration of antibiotics into the conjunctival sac, in the form of ophthalmic solutions or ointments, and steroidal or non-steroidal anti-inflammatory drugs. In chronic conjunctivitis, local antibiotic therapy should be supplemented with the use of systemic antibiotics, after prior identification of the bacteria and their antibiotic resistance. In cases of Gram-positive bacterial infections, the most commonly used topical antibiotics are fluoroquinolones, erythromycin, bacitracin, neomycin, polymyxin B and chloramphenicol, whereas Gram-negative infections are treated with aminoglycoside antibiotics [4, 11, 12].

Methicillin-resistant strains of *S. aureus* (MRSA) are known to be responsible for serious hospital infections in humans, including bloodstream infections, pyomyositis or necrotizing fasciitis, osteomyelitis, septic arthritis, Waterhouse-Friderichsen syndrome, pneumonia, and bacteraemia [13]. The extensive spread of strains susceptible to only one group of antibiotics is a serious problem [14], especially as vancomycin-resistant strains isolated from humans, have already been noted, including in Poland [15]. A very important issue is the ease of transmission of pathogens from animals to humans. Animals infected with *Staphylococcus* spp. can pose a serious threat to humans, and the prevalence of these microorganisms increases the possibility of transmission of antibiotic resistance genes among staphylococci [16, 17].

Due to the increasing drug resistance among bacterial strains, there is a need to search for alternative methods to eliminate pathogens potentially responsible for the transfer of resistance genes. One alternative method is phage therapy, using bacteriophages isolated from the environments in which specific pathogens occur.

Bacteriophages, also called bacterial viruses, are ‘natural killers’ of bacteria. They are the most abundant form of life on earth (their total number is estimated at 10^32^ virions) and are present in diverse environments (e.g. wastewater, water bodies, soil, forest undergrowth, food products, animals and humans). Bacteriophages contain only one type of nucleic acid, DNA or RNA. They also possess a capsid, which is built of structural proteins. The presence of bacteriophages is a natural mechanism that has existed for billions of years, ensuring the proper balance of various bacteria in the natural environment. These viruses show specific affinity for individual types of bacteria [18].

Bacteriophages can be lytic or temperate form. The lytic cycle of bacteriophage multiplication comprises adsorption, i.e. attachment of the phage ‘tail’ proteins to a specific receptor on the of the bacterial cell’s membrane; penetration of phage genome into the cytoplasm of the bacteria; assembly of new phages in the bacterial cells; and lysis of the cell wall. The progeny virions of the phages are released and infect additional bacteria. In the lysogenic cycle- temperate phages, not copied or expressed of DNA to make proteins, but recombines with the bacterial chromosome. In lysogenic cells, the phage exists in the form of DNA, called a prophage. Following integration with the host cell chromosome, the phage genome is lysogenized, or it may remain in the form of an episome. Lysogeny can continue for many generations, as long as the intracellular concentration of the active form of the repressor of lytic phage functions is sufficient to inhibit the transcription of early genes associated with lytic development [19].

Phages as antibacterial agents were first discovered more than 100 years ago, by Frederick Twort in 1915 and Felix d’Herelle in 1917 independently. Bacteriophages can be used to prevent and treat various bacterial infections, including zoonotic pathogens in livestock, with confirmed elimination of 99% or 100% of bacterial pathogens in poultry, cattle or pigs [20].

Each newly isolated bacteriophage is a valuable potential component of a preparation that could be used to treat bacterial infections. Given that many diseases cannot be treated using traditional methods and that the ‘new’ class of antibiotic (containing new structures and mechanism of antibacterial activity) was developed over 20 years ago, the possibility arises that we will be unable to treat infections in humans and animals.. The acquisition of ‘new’ phages is thus an important phenomenon in research centres, as not every phage meets the criteria (e.g. pH stability and lytic titre stability) for use as a component of an antibacterial preparation [21, 22].

In view of the increasing multi-drug resistance among bacteria and the need to find alternative methods to eliminate pathogens, the main purpose of this study was to assess the in vitro antibacterial effect of phages specific for *Staphylococcus* spp. strains isolated from dogs with symptoms of bacterial conjunctivitis, as an alternative to antibiotics in the elimination of infections.

## Materials and methods

### Material collection

The material was collected from dogs with clinical signs of bacterial conjunctivitis (about 120 independent cases) during standard diagnostic procedures. The animals were patients of the Department and Clinic of Animal Surgery at the University of Life Sciences in Lublin, and all samples were obtained during diagnostic procedures, such as evaluation of antibiotic resistance (antibiogram), which is essential for selecting antibiotic treatment. The owners were informed about the details of conducted clinical trials and they have given their consent. According to the present law in Poland (Experiments on Animals Act from January15th 2015, Journal of Laws of the Republic of Poland from 2015, item. 266), the study did not require the approval of the Ethics Committee. The study was performed in accordance with Directive 2010/63/EU of the European Parliament and of the Council of 22 September 2010 on the protection of animals used for scientific purposes, Chapter I, Article 1, point 5(b). Research was also approved by the Scientific Research Committee of the Department and Clinic of Animal Surgery at the University of Life Sciences in Lublin (#1/2018) concerning non-experimental clinical patients.

All samples were collected from dogs prior to treatment. Before the bacteriological examination, the patients were not administered any topical or systemic drugs.

The samples were collected from the conjunctival sac after grasping the lower eyelid to reveal the conjunctiva of the lower eyelid and third eyelid using a sterile swab (Meus s.r.i., Piove di Sacco, Italia). The sample was collected by moving the swab towards the conjunctival sac, avoiding contact with the palpebral margin and eyelashes, after which it was inoculated onto transport medium and transported for analysis within 20 min at 4 °C. The swabs were collected from the right and left eye of each dog without local anaesthesia.

Bacterial strains were isolated (about 80 isolates) on two types of media: mannitol agar (Chapman medium, BTL, PL) and Columbia blood agar with 5% sheep blood (BTL, PL) under aerobic conditions at 37 °C for 24 h. The cultures were incubated in TSB broth (BTL, PL) at 37 °C for 24 h to obtain optimum growth of pure strains. Phenotypic identification of *Staphylococcus* spp. isolates was carried out by means of Gram staining and biochemical commercial API STAPH tests. Molecular identification was carried out by MALDI-TOF MS mass spectrometry [23].

Measurements were performed with an UltrafleXtreme MALDI-TOF mass spectrometer (Bruker, Germany) equipped with a 1000 Hz neodymium-doped yttrium aluminium garnet (Nd:YAG) laser. For this method, single bacterial colonies grown on agar were re-suspended in 1.2 mL of 75% ethanol. After centrifugation at 13,000 *g* for 2 min at 20 °C and removal of the supernatant, cells were extracted with 50 μL of formic acid (Sigma-Aldrich, Poland) and 50 μL of acetonitrile (Sigma-Aldrich, Poland). After centrifugation, each of the samples was transferred onto a spot of a 384 MTP AnchorChip TF stainless steel MALDI target plate (Bruker, Germany). Then the bacterial sample was overlaid with 1 μl of matrix solution containing 10 mg/ml HCCA (a-cyano-4-hydroxycinnamic acid, Sigma-Aldrich, Poland) resolved in 50% acetonitrile and 2.5% TFA (trifluoroacetic acid, Sigma-Aldrich, Poland) and air-dried. The MALDI plate was then introduced into the spectrometer for automated measurement and data interpretation. Prior to the analyses, calibration was performed with a bacterial test standard (Bruker, Germany) containing extract of *Escherichia coli* DH5 alpha [24, 25]. The mass spectra were processed with the MALDI Biotyper 3.0 software package (Bruker, Germany), containing 3995 reference spectra corresponding to different types of bacteria.

The results were shown as the top 10 identification matches with confidence scores ranging from 0.00 to 3.00. A log (score) < 1.70 does not allow for reliable identification, a log (score) between 1.70 and 1.99 allows identification to the genus level, a log (score) between 2.00 and 2.29 means highly probable identification at the genus level and probable identification at the species level, and a log (score) > 2.30 indicates highly probable identification at the species level (according to the manufacturer’s instructions).

A catalase test the one of biochemical tests that commonly used to differentiate *S. aureus* from coagulase-negative staphylococci for a reason of virulence, was carried out to detect pathogenic Staphylococcus spp. strains. The test is performed by flooding an agar slant or broth culture with several drops of 3% hydrogen peroxide. Catalase-positive cultures bubble at once [26].

### Evaluation of the antibiotic resistance of the strains

The strains were examined to determine the profile of resistance to selected antimicrobial substances of various classes, i.e. amoxicillin (25); amoxicillin-clavulanic acid (30 μg), ampicillin (10 μg), amikacin (30 μg), ciprofloxacin (5 μg), clindamycin (2 μg); enrofloxacin (30 μg), erythromycin (15 μg), gentamicin (10 μg), kanamycin (30), methicillin (10 μg) lincomycin/spectinomycin (109 μg), vancomycin (30 μg), oxacillin (1 μg) polymyxin (300 μg), sulfamethoxazole-trimethoprim (25 μg), tobramycin (10 μg) and tetracycline (30 μg), in accordance with CLSI recommendations for these antibiotics. The antibiotic resistance profiles of the strains were determined by the disc-diffusion method on Mueller-Hinton agar (Oxoid Ltd) as described by CLSI 2017 [25] and EUCAST [27]. The MIC results were compared with values for *S. aureus* ATCC 25923.

### Preparation of the bacteriophage suspension

Bacteriophages specific for *Staphylococcus* strains were isolated and characterized in accordance with the procedure proposed by Han et al. [28], as modified by Marek et al. [29] Prior to further characterization, the phages were individually plaque-purified three times on agar plates. All of used phages were coming from our own collection and all phages were previously isolated from seawage.

Following 24 h incubation at 37 °C, the bacteriophages were collected from 0.7% agar from zones with complete lysis of bacteria (plaques) and transferred to 2 ml of TSB broth. The whole was suspended in Tryptic soy broth (TSB), with the addition of 250 μL 1 M CaCl_2_, 250 μL 1 M MgSO_4_ and 250 μL of a 3 h culture of *S. aureus*, and incubated in a shaker for 18 h at 37 °C and 120 rpm. After centrifugation at 12,000 *xg* for 30 min, chloroform was added to the supernatant to a final concentration of 2% v/v. After vortexing for 5 min, centrifugation (5000 *xg*/10 min/20 °C), and filtration, the suspension was stored at 4 °C until further analysis.

In the next stage of the study, the lytic titres of the bacteriophages were determined by the double-layer agar method on Tryptic soy agar (TSA). The range of lytic activity against the pathogens as well as the pH tolerance of the phages was determined on double-layer agar plates according to Niu et al. [30] Morphological analysis of the bacteriophages was performed by transmission electron microscopy (TEM) using slides negatively stained with 2% uranyl acetate [31]. For comparison of phage morphology, a phage specific to *S. aureus* ATCC 25923 was used as a reference.

The bacteriophage lysate was concentrated by precipitation in polyethylene glycol (PEG) 8000 solution according to Chibani-Chennoufi et al. [32]. To this end, 6.5 mL of 20% PEG 8000 NaCl buffer was added to tubes containing a suspension of bacteriophages, which was then mixed on a vortex and incubated at 4 °C for 24 h. Next, following centrifugation at 8500 *xg*/10 min/4 °C, the precipitate was suspended in a specified volume of TE buffer. Following centrifugation at 11,000 x g/10 min/4 °C, 120 μL of 20% PEG 8000 NaCl was added to the suspension, which was then incubated at 4 °C for 1.5 h. After centrifugation at 13,000 *xg*/10 min/4 °C and removal of the supernatant, Tris-EDTA (TE) buffer was added to the precipitate again. The resulting suspension was extracted with an equal amount of chloroform, followed by vortexing for 30 s to remove residual polyethylene glycol. The concentrated suspension of bacteriophages was centrifuged at 4500 x g/7 min/4 °C. The purification procedure was followed by dialysis of the bacteriophage lysate through a Pellicon membrane (1000 kDa, EMD Millipore) according to Szermer-Olearnik and Boratynski [33].

The aqueous phase was collected, and following determination of the lytic titre, was stored at 4 °C until use as a component of eye drops.

The number of bacteriophage plaque-forming units (PFU) was determined by serial dilutions of the phage lysate suspended in the above-mentioned solution prepared for eye drops [34].

### Preparation of eye drops and assessment of their antibacterial efficacy in vitro

Only phages with strong lytic titres > 10^8^ PFU/mL and with stabilized lytic properties were used to prepare eye drops. Eye drops were prepared using eight different solutions (Table 1).

**Table 1:** The Percentage of Individual Experimental Variants of Eye Drops for Dogs with Bacterial Conjunctivitis

Components	Formulations							
	1	2	4	7	8	9	10	12
The bacteriophages suspension 10^−10^ PFU/mL	20.0% v	20.0% v	20.0% v	20.0% v	20.0% v	20.0% v	20.0% v	20,0% v
Boric acid	1.1% w	–	–	–	–	–	–	–
Sodium tetraborate	0.29% w	–	–	–	–	–	–	–
Sodium chloride	0.29% w	0.9% w	0.9% w	0.9% w	–	–	–	–
Mannitol	–	–	–	–	5.0% w	–	–	–
85% Glycerol	–	–	–	–	–	2.42% v	2.42% v	3,97% v
Disodium EDTA	–	0.05% w	0.05% w	–	0.05% w	–	0.05% w	0,05% w
Benzalkonium chloride	–	–	0.01% w	0.01% w	–	–	–	0,01% w
0.2 M NaOH	–	q.s. to 7.05	q.s. to 7,07	q.s. to 6,93	q.s. to 6.92	–	q.s to 7.02	q.s to 6,92
Water for injection	to 100% v	to 100% v	to 100% v	to 100% v	to 100% v	to 100% v	to 100% v	to 100% v
pH	7.52	7.05	7.07	6.93	6.92	7.06	7.02	6,92
Osmotic pressure mOsm/kg	290	280	285	286	292	310	287	517 mOsm/kg

Under aseptic conditions, the solids listed in Table 1 were dissolved in Aqua Pro Injectione (Baxter, PL). Depending on the composition of the formulation, glycerol was added and the pH of the solution was adjusted to 6.92–7.52 using 0.2 N sodium hydroxide solution. A suspension of bacteriophages was then added and supplemented with water where necessary to 100 mL. The solutions were mixed and then filtered through a Schott G-5 glass filter.

The experimental phage formulations prepared in this manner were then placed in hermetically sealed 10 mL dark glass bottles and stored at 4–8 °C in a refrigerator. The pH of the solutions was measured with a CP-411 pH-meter (Elmetron), and the osmotic pressure with a Trident 800 cL osmometer. The percentage composition of each formulation is presented in detail in Table 1.

Of the eight eye drop variants, only those that did not significantly affect the viability and lytic titre of the bacteriophages specific for *Staphylococcus* spp. strains were used for further in vitro testing.

### Limulus Amebocyte lysate assay for endotoxins estimation

For quantification of the cytotoxicity of bacterial endotoxins in all experimental eye drops, the Chromogenic Limulus Amebocyte Lysate (LAL, Lonza) test was performed. The procedure was carried out on pyrogen-free microplates according to the manufacturer’s instructions.

Prior to analysis, the samples were diluted with Binding Buffer. First all samples were incubated on microassay plates overnight at room temperature with shaking. For this purpose, 50 μL of sample was dispensed in duplicate in a 96-well flat-bottomed plate. The blank wells contained 50 μL of water (LAL Reagent Water; Lonza) instead of sample. Then 50 μL of LAL was added to all microplate wells. After 10 min of incubation at 37 °C, 100 μL of pre-warmed substrate solution was mixed with each of the LAL-samples and incubated at 37 °C for an additional 6 min. The reaction was stopped with 100 μL of stop reagent. If endotoxin was present in the sample, a yellow colour appeared. The absorbance was determined with a BioRad 680 model microplate reader at a wavelength of 405–410 nm. The concentration of endotoxins was calculated from a standard curve and shown in European units (EU) [33].

Every 7 days the phage compositions were tested for antibacterial activity and lytic titre stability by the double-layer agar plate method [32].

The stability of the antibacterial titre was evaluated for preparations stored in sealed bottles as well as after opening and reclosing. The efficacy of the eye drops against *Staphylococcus s*pp. strains was determined in in vitro conditions.

## Results

All dogs in the study had conjunctivitis characterized by conjunctival hyperthermia and purulent ocular discharge. The clinical signs of the disease were conjunctival hyperaemia and purulent discharge from the conjunctival sac. Swabs collected from dogs confirmed suffering from purulent conjunctivitis, which in 35 cases was associated with keratoconjunctivitis sicca and in 48 cases with follicular inflammation of the third eyelid, while in 18 cases it was primary bacterial conjunctivitis. In 19 dogs, purulent inflammation was accompanied by chronic superficial keratitis. The dogs had no systemic diseases.

In our study, we isolated the bacteria towards Staphylococcus spp. The tests confirmed the occurrence of *Staphylococcus* spp. strains as etiological agents of bacterial conjunctivitis in the animals. In total, 80 *Staphylococcus* spp. strains were isolated.

The *Staphylococcus* species represented in the highest numbers was *S. epidermidis* (*n* = 45), followed by *S. aureus* (*n* = 25), while *S. pseudintermedius* was the least numerous (*n* = 10), as confirmed by both biochemical tests and MALDI TOF mass spectrometry.

MALDI TOF mass spectrometry analysis confirmed the high percentage of strain identification at the species level. The results are shown in Table 2.

**Table 2:** Mean log (score) results of MALDI-TOF MS analysis of *Staphylococcus* spp. isolated from dogs with bacterial conjunctivitis

Log (score)	Description	Symbol	Bacterial species		
			*S. aureus* n = 25	*S. pseudintermedius* n = 10	*S. epidermidis* n = 45
2.300–3.000	Highly probable identification at the species level	++++	12	7	39
2.000–2.299	Highly probable genus identification and probable species identification	+++	7	2	4
1.700–1.999	Identification to the genus level	++	4	1	2
< 1.700	Does not allow for reliable identification	+	1	0	0

Analysis of the antibiotic resistance of the bacterial strains showed that a high percentage of strains were resistant to more than one antibiotic. All strains tested were resistant to erythromycin, tetracycline and oxacillin, and all *S. pseudintermedius* strains were also resistant to lincomycin/spectinomycin and gentamicin (Table 3). A high level (63.75–76.25%) of multi-drug resistance was observed, i.e. resistance to least 3 of the 18 antibiotics (Table 3). Additionally, 100% of *S. aureus* strains were shown to be resistant to ampicillin and kanamycin. It is worrying that among the isolates tested, 3 strains of *S. aureus* showed resistance to vancomycin (Table 3). However, these results for vancomycin were obtained only by the disc-diffusion method, while the MIC test did not show resistance to vancomycin.

**Table 3:** Antibiotic resistance profiles of Staphylococcus species isolated from dogs with bacterial conjunctivitis

Antibiotic	Numbers and % of resistant and susceptible bacteria						Total % of resistance
	*S. epidermidis* (n = 45)		*S. aureus* (n = 25)		*S. pseudintermedius* (n = 10)		
	R	S	R	S	R	S	
Amoxicillin AML25	26.6	73.4	76	24	20	80	41.5
Amoxicillin/clavulanic acid AMC30	6.6	93.4	24	76	0	100	11.25
Ampicillin AMP10	8.9	91.1	**100**	**0**	20	80	38.75
Amikacin AN 30	71.1	28.9	60	40	40	60	63.75
Enrofloxacin ENR5	22.2	77.8	8	92	30	70	18.75
Lincomycin/spectinomycin LS109	0	100	0	100	**100**	**0**	12.5
Sulfamethoxazole/trimethoprim SXT25	80	20	72	28	70	30	76.25
Polymyxin PB 300	0	100	0	100	0	100	0
Erythromycin E 15	**100**	**0**	**0**	**0**	**100**	**0**	**100**
Gentamicin CN 10	15.5	84.5	12	88	**100**	**0**	25
Tetracycline TE	**97.7**	2.3	**100**	**0**	**100**	**0**	**100**
Clindamycin DA 2	6.66	93.34	52	48	0	10	20
Oxacillin OXA 1	**10**	**0**	**100**	**0**	**100**	**0**	**100**
Vancomycin VA 30	0	100	12	88	0	10	3.75
Tobramycin TOB 10	55.5	44.5	36	64	**70**	**30**	51.25
Kanamycin K 30	66.7	33.3	**100**	**0**	40	60	73.75
Ciprofloxacin CIP 5	0	100	4	96	0	100	1.25
Methicillin MEL 10	2.2	97.8	12	88	0	100	32.5

Legend: Numbers in parentheses indicate percentages of strains

The highest percentage of strains susceptible to the antibiotics was observed for *S. pseudintermedius*, in which 100% of strains were susceptible to 6 of 18 antibiotics: amoxicillin/clavulanic acid, polymyxin, clindamycin, vancomycin, ciprofloxacin and methicillin. *S. epidermidis* strains were 100% susceptible to 4 antibiotics, i.e. lincomycin/spectinomycin, polymyxin B, vancomycin, and ciprofloxacin, while 100% of *S. aureus* strains were susceptible only to lincomycin/spectinomycin and polymyxin B. It should be emphasized that all three staphylococcal species were 100% susceptible to polymyxin B (Table 3).

**Fig. 1:**
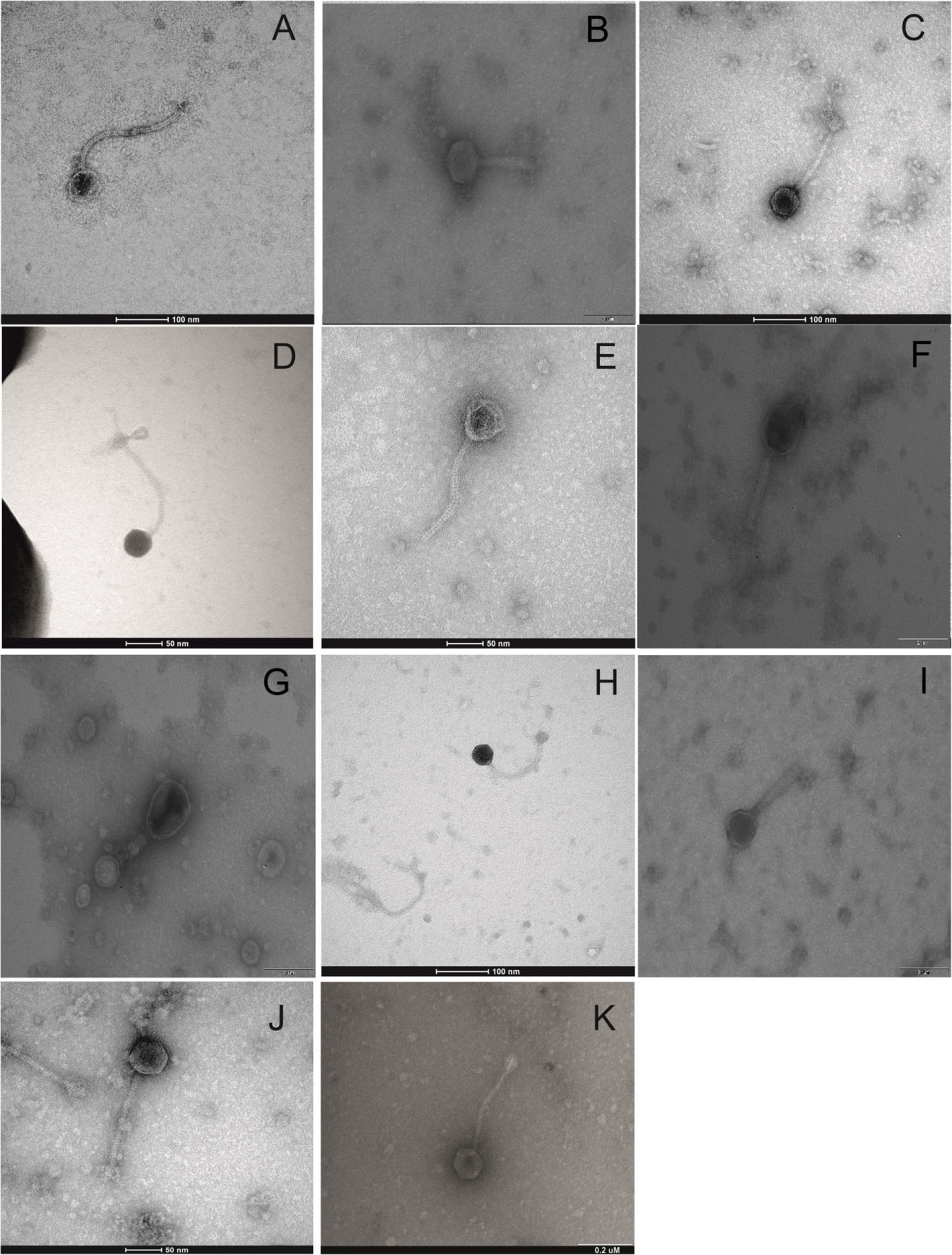
Negative-stained electron micrographs of bacteriophages induced in isolates of *Staphylococcus* spp. Legend: Myoviridae and Siphoviridae phages: A - phage no. W28, B - W15, C - W36, D - W29B, E - W29, F - W17, G - W31, H – W1, I – W33, J - W27; K- Reference Myoviridae phage specific for *S. aureus* ATCC 25923

We obtained 10 bacteriophages specific for pathogenic *Staphylococcus* spp. isolated from dogs. All tested phages were from our own collection and were isolated from water samples. All information about the phages was included in Patent Applications P.427797 and 427798 [35, 36]. The results of transmission electron microscopy (TEM) analysis showed that most of the *Staphylococcus* spp. phages under study belonged to the *Myoviridae* family, based on icosahedral heads with sizes ranging from 64 to 95 nm and long contractile tails from 190 to 250 nm in the extended state (Fig. 1). The lytic titres were estimated as 10^6^–10^10^ PFU/mL. Most of the phages expressed lytic properties against more than 80% of tested *Staphylococcus* spp. strains in our collection, but only 5 phages (W15, W17, W33, W31 and W36) showed long-term stability in antibacterial activity against all examined strains. These phages were used as a cocktail in the eye drops (Table 4).

**Table 4:** Types, titres and lytic activity spectrum of bacteriophages specific for *Staphylococcus* spp. isolates obtained from dogs with bacterial conjunctivitis

Phage no.	Morphology	Bacterial host	Lytic titre PFU	Spectrum of lytic activity: total percentage of *Staphylococcus* spp. strains undergoing lysis
W28	*Siphoviridae*	*S. aureus*	10^10^	68 (85%)
W15	*Myoviridae*	*S. aureus*	10^10^	80 (100%)
W36	*Myoviridae*	*S. epidermidis*	10^9^	80 (100%)
W29B	*Siphoviridae*	*S. epidermidis*	10^8^	72 (90%)
W29	*Siphoviridae*	*S. pseudintermedius*	10^6^	68 (85%)
W17	*Myoviridae*	*S. epidermidis*	10^10^	80 (100%)
W31	*Myoviridae*	*S. aureus*	10^9^	80 (100%)
W33	*Myoviridae*	*S. pseudintermedius*	10^10^	80 (100%)
φW1	*Siphoviridae*	*S. epidermidis*	10^9^	80 (100%)
φW27	*Siphoviridae*	*S. aureus*	10^7^	75 (93.7%)

**Fig. 2:**
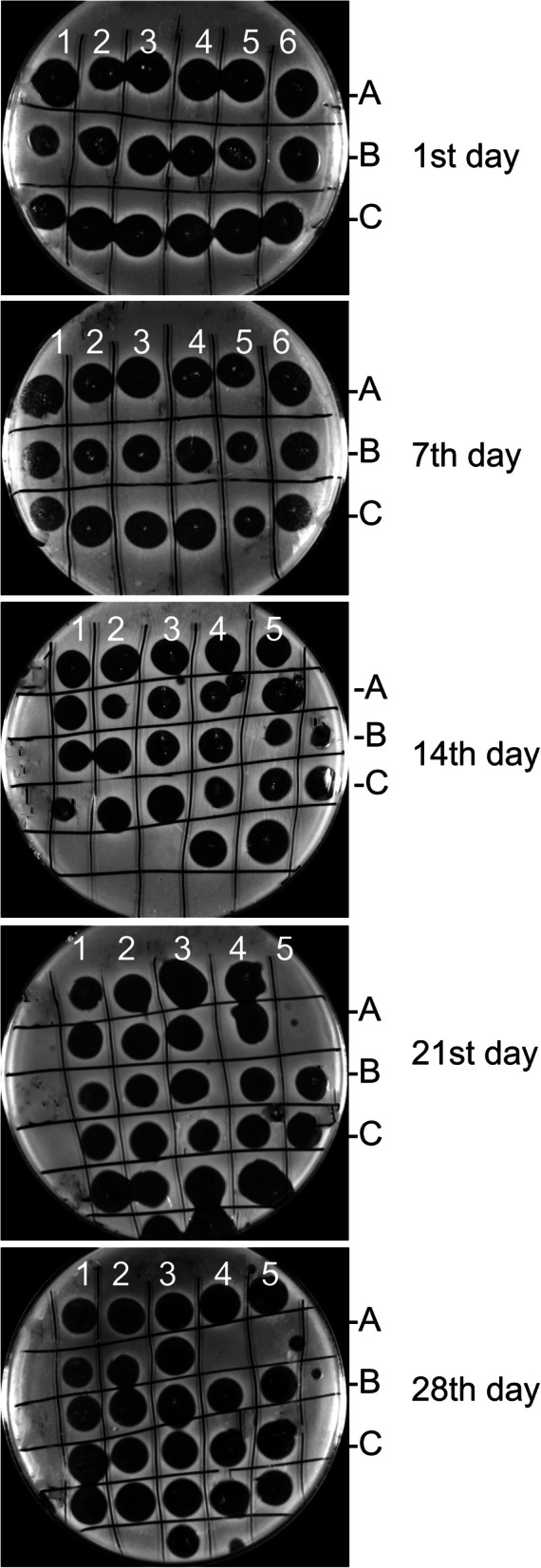
In vitro lytic activity of experimental eye drops against selected strains of *Staphylococcus* spp. Legend: **a** Experimental eye drops solution no 4; **b** Experimental eye drops solution no 7; C - experimental eye drops solution no 12 Arabic numbers 1 to 6 refer to dilutions of experimental phage drops [1- stock solution, 2- dilution 1:2, 3- dilution 1:4, 4- dilution 1:8; 5- dilution 1:16; 6- dilution 1:32].

Only three of the variants of eye drop solutions, nos. 4, 7 and 12, had no negative impact on the bacteriophages (destruction or significant reduction of lytic titre). For this reason, these solutions were selected for further analysis, to assess their bactericidal efficacy against *Staphylococcus* spp. test strains obtained from dogs with symptoms of bacterial conjunctivitis. Based on their antibacterial properties and the time during which a high antibacterial titre persisted, eye drop solutions 4, 7 and 12 were confirmed to be highly effective (see results in Fig. 2). Figure 2 presents examples of the antibacterial effect of the experimental drops, but only for dilutions resulting in a complete lysis zone in in vitro conditions. The eye drops remained active, as evaluated by storing the suspension at 4–8 °C in unopened dark bottles, for nearly 6 weeks.

The level of endotoxin in the eye drop solutions estimated in the Limulus amoebocyte lysate assay was below 50 EU/mL.

The results confirmed the significant antibacterial effect of the experimental eye drops containing bacteriophages. All bacterial strains were destroyed after the drops were applied to the bacterial cell cultures on double-layer agar plates. Formulations 7 and 12 maintained their constant antibacterial titre of ≥10^8^ PFU/mL for 21 days after the bottles had been opened and closed again, The corresponding period for formulation no. 4 was shorter, at 14 days, after which the lytic titre fell significantly and was insufficient to ensure elimination of bacteria in vitro. The bottles with the ophthalmic solutions were stored at 4–8 °C. We did not detect any changes in the pH of the ophthalmic solutions, which corresponded to pharmacopoeial values (the pH range for eye drops according to Pharmacopeia European X, Ph. Eur is 3.5–8.5), while the osmotic pressure slightly decreased, but was still within ocular tolerance up to the end of the study (United States Pharmacopeia USP XIII).

Patent applications were submitted for two phage formulations in the form of eye drops to the Patent Office of the Republic of Poland in 2018 – application nos. P.427797 and P.427797 [35, 36].

## Discussion

The results of the research confirmed a high percentage share of strains of the genus *Staphylococcus* spp. as etiological agents of bacterial conjunctivitis in dogs. Particularly noteworthy is the high number (45) of strains of the species *S. epidermidis*, representing over 56% of all bacterial isolates. The number of *S. aureus* isolates was 25, accounting for 31%. The results differ from those reported by Junior et al. [37], in which the percentage of *Staphylococcus* strains isolated from cases of bacterial conjunctivitis in dogs was > 66%, of which *S. epidermidis* isolates constituted only 6%. *S. aureus* accounted for 25% of strains, similar to the percentage obtained in our study (31%). However the % of the prevalence of *S. epidermidis* in dogs was similar like in the study of Gómez-Sanz et al. [38], where the total % of prevalence of *S. epidermidis* was 66%. The high percentage of *S.epidermidis* strains observed in our as well as the cited research may be result from the presence of specific isolates in a given area, region or environment. However in case of the rest *Staphylococcus *spp. strains the obtained results were very similar to cited authors.

Our research confirmed the high rate of resistance of *Staphylococcus* spp. strains to more than one antibiotic – in many cases to a third of the antibiotics used in the study.

The resistance of the test strains to selected antibiotics observed in the present study supports results obtained by other authors, such as Junior et al. [37] , who reported 100% resistance to sulphonamide and 91.67% resistance to tetracycline for *S. aureus*, and 75% resistance to tetracycline and ceftriaxone for *S. intermedius*. The latest research indicates a significant upward trend in resistance to β-lactams, including oxacillin, as well as erythromycin and tetracycline [38].

It is concerning that 100% of the tested isolates were resistant to erythromycin, which in addition to fluoroquinolones and chloramphenicol is commonly used to treat bacterial conjunctivitis in dogs [4]. Infections caused by Gram-negative rods are additionally treated with aminoglycoside antibiotics [39].

The results of our study, showing a high percentage of resistant bacteria, confirm the need to look for new therapeutic solutions as alternatives to antibiotics to eliminate the etiological agents of bacterial conjunctivitis in dogs. A positive result observed in the presented own studies is the 100% sensitivity of all tested *Staphylococcus* spp. isolates to Polymixin B.

The five *Myoviridae* phages (W15, W17, W33, W31 and W36) used in the present study as components of experimental eye drops were selected for their wide spectrum of infectivity and lytic nature. According to some authors, virulent bacteriophages of the *Myoviridae* family specific for *Staphylococcus* spp. may be better suited for phage therapy due to their lack of or highly restricted capacity for horizontal gene transfer, as in the case of lysogenic phages. Other phages like *Siphoviridae* family, are mostly the temporary phages and can be reservoirs of antibiotic resistance genes that can be transferred to bacteria on during the lysogeny process in bacteria [20].

However, as with antibiotics, bacteria have the potential to acquire resistance to bacteriophages. Bacteria could induce resistance mechanisms, through a change or loss of surface receptors, secretion of substances that prevent phage adhesion to the bacterial cells, activation of measures for blocking phage DNA injection into the cell, or inhibition of phage replication and release [40]. According to a review by Rohde et al. [41], the mechanism of antibacterial resistance developed by phages is mainly observed in in vitro studies. For example, it is correlated with the appearance of new anti-phage spacers in CRISPR loci (clustered regularly interspaced short palindromic repeats). Also, because the total number of bacteriophages in the environment significantly exceeds the number of bacteria, thus far no such significant increase in bacteria resistant to phages has been observed.

The experimental phage preparations developed in the study in the form of eye drops exhibited 100% efficacy in vitro against all tested *Staphylococcus* isolates. Particularly noteworthy is the long duration of activity and constant lytic titre of preparations 7 and 12, both after the bottle had been opened (min. 21 days) and in the case of hermetically sealed packaging (min. 28 days) and refrigeration at 4–8 °C. This is advantageous, as the shelf life of antibiotic eye drops in a pharmacy without a preservative is up to 24 h after opening, and up to 10 days with the addition of a preservative. Examples of this type of preparation used in the treatment of eye diseases include compounded eye drops containing detreomycin or gentamicin. Hence the experimental phage preparations in the form of eye drops have a significantly longer shelf life, which is an argument in their favour.

Given the high sensitivity of the eye to damage and external factors, eye drops must meet several requirements so as not to cause irritation or sensitization of the eye. They must be sterile, isotonic with tear fluid, and have a pH in the range of 3.5–8.5. To meet these requirements, eye drops may contain auxiliary substances such as isotonizing and buffering agents, preservatives, and viscosity modifiers. These substances must not exert their own pharmacological or irritating effects in the amounts used. In the experimental eye drops used in this study, preservatives were used to stabilize the bacteriophages and extend the shelf-life of the preparations. The most widely used preservative in eye drops is benzalonium chloride, in concentrations ranging from 0.005 to 0.2%. BAK toxicity depends on the amount administered daily, the duration of the treatment, and its concentration in the solution. However, at a concentration of 0.01%, short-term administration of this agent should not be irritating, as confirmed in many studies [42, 43].

Despite the 100% antibacterial effect demonstrated in vitro, the results represent only the first stage of research and require continuation as experimental therapy in vivo, which will be the subject of further study.

## Conclusions

The 100% antibacterial effect of the experimental eye drops containing a mixture of five phages specific for *Staphylococcus* spp. strains isolated from dogs with symptoms of bacterial conjunctivitis indicates that research could be undertaken on the use of bacteriophages to treat infections caused by *Staphylococcus* spp. strains in companion animals. If positive clinical effects are obtained in animals, the results can be used in applied research as potential components of antibacterial preparations for humans and animals, available for use in national and international studies. It is worth emphasizing the innovative nature of the research aimed at finding alternative solutions for eliminating multi-drug resistant pathogens.

## Data Availability

The datasets used and/or analysed in the study are available from the first and corresponding author on reasonable request. Some of the materials are available as patent applications UPRP P.427797 and UPRP P.427797 (2018).

## Abbreviations

CaCl_2_: Calcii chloridum
EDTA: Ethylenediaminetetraacetic acid
HCCA: A-cyano-4-hydroxycinnamic acid
LAL: Limulus Amoebocyte Lysate
MgSO_4_: Magnesium sulphate
MIC: Minimum inhibitory concentration
MRSA: Methicillin-resistant *Staphylococcus aureus*
NaCl: Sodium chloride
PEG: Polyethylene glycol
PFU: Plaque-forming unit
TE: Tris EDTA Buffer
TEM: Transmission electron microscopy
TSA: Tryptic Soy Agar
TSB: Tryptic Soy Broth
μL: Microlitre
